# Novel blood lncRNA biomarkers associated with clinical severity and specific cognitive dimensions in Alzheimer's disease

**DOI:** 10.1177/13872877261470430

**Published:** 2026-07-29

**Authors:** María I. Mosquera-Heredia, Oscar M. Vidal, Ernesto Barceló, Luis C. Morales, Carlos Silvera-Redondo, Daniel A. Bolívar, Ricardo Allegri, Mauricio Arcos-Burgos, Pilar Garavito-Galofre, Jorge I. Vélez

**Affiliations:** 1 28003 Universidad del Norte, Barranquilla, Colombia; 2 Instituto Colombiano de Neuropedagogía, Barranquilla, Colombia; 3 Department of Health Sciences, Universidad de La Costa, Barranquilla, Colombia; 4 Grupo Internacional de Investigación Neuro-Conductual (GIINCO), Universidad de La Costa, Barranquilla, Colombia; 5 Institute for Neurological Research 58782 FLENI, Buenos Aires, Argentina; 6 Grupo de Investigación en Psiquiatría (GIPSI), Departamento de Psiquiatría, Instituto de Investigaciones Médicas, Facultad de Medicina, Universidad de Antioquia, Medellín, Colombia

**Keywords:** Alzheimer's disease, biomarkers, exosomes, long non-coding RNA, neuropsychological tests, predictive power score

## Abstract

**Background:**

Differential expression of long non-coding RNAs (lncRNAs) in brain, serum, and blood show strong potential to distinguish Alzheimer's disease (AD) from healthy controls.

**Objective:**

To explore whether lncRNA signatures delineate AD pathology and map to distinct, multidimensional cognitive domains, enhancing specificity in assessing AD severity and progression.

**Methods:**

We profiled 29,603 lncRNAs transcripts in blood samples from 15 AD patients and 15 healthy controls, alongside comprehensive neuropsychological assessments. Generalized Linear Models and Predictive Power Score analyses, with statistical prioritization, identified lncRNAs associated to AD neuropsychological architecture.

**Results:**

Several lncRNAs share strongly associated with cognitive performance and AD severity, mapping to genes involved in key AD-related molecular processes, including synaptic and neurotransmitter regulation (e.g., *EPHB1*, *CHRNA4*, *TEAD1*), protein homeostasis and Aβ pathology (e.g., *FBXL2*, *FAM221A*, *APP*), mitochondrial function and cellular stress (e.g., *VDAC3*, *PPT2-EGFL8*), neuroinflammation and immune regulation (e.g., *TEAD1*, *EMX2OS*, *LY6E-DT*), epigenetic and transcriptional control (e.g., *PRDM2*, *DLEU1*, *FIRRE*), neuronal excitability (e.g., *KCNJ14*), and neuroprotection and synaptic plasticity (e.g., *SIL1*). Novel associations included ferroptosis, DNA stability, microtubule dynamics, and dendritic orientation (e.g., *BTB3*, *DICER1*, *GNG7*, *IBA57*, *NEAT1*, *POT1*, *SRD5A3*).

**Conclusions:**

We identify candidate lncRNA signatures that may serve as potential biomarkers and enhance our understanding of the molecular basis of the cognitive architecture in AD, opening new avenues for biomarker identification and targeted therapeutic strategies development. Validation in larger, diverse cohorts is essential to confirm their mechanistic contributions to AD.

## Introduction

Alzheimer's disease (AD) is a neurodegenerative disorder of complex etiology characterized by cognitive decline, memory impairment.^1^ Recognized as the leading cause of dementia worldwide, AD neuropathological hallmarks are the brain amyloid-β (Aβ) plaques deposition and neurofibrillary tangles development, which disrupt neuronal communication and cause cell death in the end.^2^ Despite extensive research, the exact AD neuropathological mechanisms are still not fully understood, and therapeutic approaches are ineffective.

Long non-coding RNAs (lncRNAs) are RNA transcripts >200 nucleotides that do not code for proteins.^3^ Unlike messenger RNAs (mRNAs), which serve as templates for protein synthesis, lncRNAs perform regulatory functions including cell differentiation and development, responses to environmental stimuli,^1^ and play a significant role in AD pathogenesis by modulating Aβ production, tau hyperphosphorylation, and neuroinflammation.^4^

The lncRNAs can influence synaptic plasticity, which is crucial for learning and memory.^5^ A recent study identified that lncRNA CASC15 enhances learning and memory in mice,^6^ suggesting that dysregulation of lncRNAs involved in synaptic plasticity may contribute to cognitive deficits.^5^ Additional research showed that the AD cognitive severity correlates with specific lncRNAs expression levels, indicating a potential link between lncRNAs molecular changes and clinical manifestations.^7^ Furthermore, the disruption of brain-subregion-specific lncRNA-patterns, are associated with AD.^8^

Other lncRNA signatures such as BC200 and NEAT1 have emerged as potential AD diagnostic biomarkers for AD.^4,9,10^ The lncRNA NEAT1 may play a role in the pathogenesis of AD by promoting mitochondrial dysfunction.^11^ Depletion of NEAT1 has been shown to rescue mitochondrial dysfunction in animal models of AD^11^ and is upregulated in AD and other neurodegenerative diseases, suggesting a potential role in the common pathological mechanisms.^12^ Similarly, the plasma lncRNA 51A levels significantly correlated with AD status (versus healthy controls) in a positive direction, while negatively correlated with Mini-Mental State Examination (MMSE) scores, insinuating that higher 51A lncRNA levels are associated with both AD status and cognitive impairment severity.^13^

The BACE1-AS is significantly upregulated in AD brains, where it functions by stabilizing BACE1 lncRNA, which increases the production of Aβ peptides, including the pathogenic Aβ_42_ isoform.^14,15^ This upregulation likely contributes to the accumulation of amyloid plaques characteristic of AD pathology.^16^ Thus far, elevated plasma levels of exosomal BACE1-AS have been proposed as a promising biomarker for early AD detection.^17^ This finding is of particular importance because in AD animal models, the experimental knockdown of *BACE1-AS* improves memory and learning behaviors, pointing out this molecule as a potential therapeutic target.^17^

Neuroinflammation also plays a critical role in the pathogenesis of AD.^18^ Chronic inflammation in the brain contributes to neuronal damage and cognitive decline.^19^ lncRNAs can regulate neuroinflammatory responses by modulating microglial activation and cytokine production,^20^ and can affect AβPP processing and Aβ production.^16^ The knockdown of RN7SL1, an RNA molecule with a role in the immune cell function, revealed that a distinctive RN7SL1 lncRNA pattern improves and modulate microglial responses and reduce proinflammatory cytokine production.^20^

Furthermore, lncRNAs function as epigenetic regulators in AD by recruiting chromatin-modifying enzymes,^21^ and interacting with chromatin remodeling complexes and the dysregulation of this function, correlated with cognitive decline and memory impairment.^22^ It appears that AD-associated lncRNAs contribute to epigenetic mechanisms-including histone deacetylase-mediated tau pathology, amyloid plaque accumulation,^8^ and DNA methylation patterning guided by lncRNAs to specific genomic loci.^21^ However, the systemic role of lncRNAs in AD-related DNA methylation changes remains to be studied.^21,23^ Finally, lncRNAs can act as competing endogenous RNAs (ceRNAs) by binding to microRNAs (miRNAs)^24^ to regulate the expression of miRNA target genes.^25^ This lncRNAs-associated-ceRNA-network disfunction showed an association with the development of AD.^26^

Thus far, the identification of brain lncRNAs function will provide insights into hidden mechanisms underpinning AD onset and progression. A meta-analysis found that lncRNAs exhibited high accuracy in identifying individuals affected with AD from controls using blood samples.^27^ This knowledge will support and improve AD diagnostic, prognostic, and eventually, the definition of therapeutic targets and biomarkers.

In a previous publication, we explored the role of mRNA in the neuropsychological architecture of AD.^28^ To follow these findings, here we tackle the hypothesis that exosome-derived lncRNA signatures correlate with the multidimensional the neuropsychological architecture of AD. Our analysis of lncRNAs seeks to identify expression patterns that correlate with specific cognitive and behavioral domains in AD, with the goal of pinpointing potential biomarkers for neuropsychological deficits and bridging molecular insights with clinical progression to enable non-invasive monitoring and personalized therapeutic strategies.

## Methods

### Participants

We enrolled 30 participants (15 AD patients, 15 healthy controls) from the Instituto Colombiano de Neuropedagogía (ICN), Barranquilla, Colombia. All participants underwent a standardized clinical diagnostic pathway. Participants underwent clinical evaluations, family history assessments, neurological/neuropsychological examinations, and structured interviews. Eligibility for the study was determined using the Montreal Cognitive Assessment (MoCA)^29^ and the published inclusion criteria.^28,30^

All individuals were >65 years of age, met the diagnostic criteria for AD according to the Diagnostic Statistical Manual (DSM) version V (DSM-V)^31^ and had a MMSE^32^ between 0 and 18 points. Individuals within this group with familial AD, the presence of other neurological disease, major psychiatric diseases, and psychoactive substance use or excessive alcohol consumption were excluded.^28,30^ Structural neuroimaging (magnetic resonance imaging, MRI) was used in all cases to assess atrophy patterns and rule out other structural or vascular causes of cognitive impairment.

Etiologic assignment was performed by a multidisciplinary team from the ICN based on clinical history, cognitive profiles, and structural brain imaging (MRI) to exclude secondary causes. Standardized biomarkers such as amyloid/tau positron emission tomography (PET) or cerebrospinal fluid (CSF) Aβ/tau were not available for this cohort. Finally, an electroencephalogram was performed to all participants. Additional data for each participant such as age at the beginning of the study, sex, educational level, marital status, weight and height were also recorded through the clinical history. In all participants diagnosed with AD, the age of onset (AOO) of the disease was defined as the age at onset of symptoms according to previous research.^33,34^ AOO was determined during anamnesis with information provided by the patients or their relatives, and by seeking confirmation from various sources, such as the neurologist's assessment and neuropsychological evaluations. This strategy has been shown to be very accurate.^35^

Individuals in the control group corresponded to healthy non-family volunteers aged >70 years without suspected AD recruited from non-family volunteers, and a MMSE between 19 and 29 points. In this group, individuals with depression, mild cognitive impairment (MCI) or dementia, the presence of any neurological disorder, major psychiatric illnesses and using of psychoactive substances or excessive alcohol consumption were excluded.^28,29,30^

### Neuropsychological assessment

All participants underwent comprehensive neuropsychological evaluation as previously described elsewhere,^28,30^ which included the following tests: The MoCA,^36^ The Boston Naming Test (BNT),^37,38^ Rey-Osterrieth complex figure,^39^ Rey Auditory Verbal Learning Test,^40^ Trail Making Test (TMT),^41,42^ Symbol Digit Modality Test,^43^ Stroop color and word test,^44^ Token Test,^45^ Benton's Visual Retention Test,^46^ Clock Drawing test,^47^ The Memory Scale subtest of the Wisconsin Card Testing Test,^48^ The Geriatric Depression Screening Test,^49^ the Global Deterioration Scale,^50^ the Barthel Functional Index^51^and the Neuropsychiatric Inventory.^52^ Finally, an electroencephalogram was performed to all participants. Additional data for each participant (i.e., age at the beginning of the study, sex, educational level, marital status, weight and height) were also recorded.

Missing data, a common feature in clinical studies of AD, were imputed using the missForest^53,54^ package for R.^55^ Subsequent statistical analyses were performed on the imputed data set. The study received ethical approval from Universidad del Norte (Acts #188 of May 23, 2019 and #198 of October 31, 2019).

### RNA isolation and extraction, and lncRNA microarray study

Blood samples were collected to isolate circulating exosomes following our previously established protocol.^28^ Serum was obtained from 6 mL of whole blood via centrifugation (4000 RPM, 10 min), followed by a secondary clearing step (5000 RPM, 30 min) to remove cellular debris. Exosomes were isolated from 1000 µL of cleared serum using the Total Exosome Isolation Reagent (catalog #4478360, Thermo Fisher Scientific, USA) following the manufacturer's protocol with optimized modifications. Briefly, samples were incubated with the reagent at −20°C for 30 min, followed by centrifugation at 10,000 RPM for 30 min. The resulting pellet was resuspended in 200 µL of 1X PBS. Exosomal morphology and size were validated via scanning electron microscopy after encapsulation with nanodiamond particles; measurements confirmed a maximum diameter of 160 nm^28^.^28^

RNA was extracted using a standardized acid phenol-chloroform protocol. Resuspended exosomes were treated with a 2X denaturing solution and 400 µL of acid phenol:chloroform, followed by phase separation at 12,000 RPM for 13 min. The aqueous phase was precipitated overnight at −20°C using 80% isopropanol and 3 M sodium acetate (pH 5.2). After centrifugation and 75% ethanol washes, the RNA pellet was resuspended in 50 µL of RNase-free water and treated with DNase I (catalog #EN0521, Thermo Fisher Scientific, USA). RNA concentration and purity (OD 260/280 and 260/230 ratios) were quantified using a NanoDrop 2000 (Thermo Fisher Scientific, USA).^28^

Comprehensive lncRNA expression profiling of 30 samples (15 AD cases, 15 controls) was performed by Arraystar, Inc. (Rockville, MD, USA; https://www.arraystar.com/). RNA integrity was verified via denaturing agarose gel electrophoresis (28S:18S ratio ∼2:1). Samples were amplified and labeled with Cyanine-3 (Cy3) using the Arraystar Flash RNA Labeling Kit via a random priming method. Labeled cRNA was purified (RNeasy Mini Kit, Qiagen) and quantified; only samples meeting the thresholds of >1.65 μg yield and >9 pmol Cy3/μg specific activity proceeded to hybridization. Labeled cRNA (1 μg) was fragmented and hybridized onto the Arraystar Human LncRNA Microarray v5.0 for 17 h at 65°C. This platform targets 39,317 lncRNAs (8393 gold standard; 30,924 reliable). Arrays were washed and scanned using an Agilent G2505C scanner.^28^

Raw signals were extracted using Agilent Feature Extraction (v11.0.1.1). Data were quantile-normalized and adjusted using a linear model in GeneSpring GX v12.1. Differentially expressed lncRNAs (DELs) were identified based on a Fold Change (FC) ≥ 1.5 and a false discovery rate (FDR)-corrected *p*-value < 0.05 (two-sample *t*-test). Functional annotation and localization were performed using multiple databases, including GENCODE v21 (https://www.gencodegenes.org), LncBook (https://ngdc.cncb.ac.cn/lncbook/), and AlzGene (http://www.alzgene.org/).^28^

### lncRNAs linked to neuropsychological manifestations of AD

lncRNAs correlated to neuropsychological manifestations of AD were identified using generalized linear models (GLMs).^56^ For each neuropsychological variable *y_i_* (i = 1, 2,…, 25), a GLM of the form *y_i_* ∼ lncRNA*_j_* + AD + Age + Sex + Schooling was fitted, where lncRNA*_j_* corresponds to the expression of the *j*th lncRNA (*j* = 1,2,…, 29,603), AD is a binary diagnosis indicator, Age is the age of the individual at the beginning of the study and Schooling is the years of education. The family distribution, a main component of a GLM, was selected according to the nature of the neuropsychological variable. Thus, neuropsychological variables representing counts were modelled using a Poisson distribution and those of continuous nature were modelled using a Gamma distribution to account for potential skewness. The regression coefficient ${\hat{\beta }}_{j}$ associated with lncRNA*_j_* was extracted with its standard error ${\hat{\mathrm{SE}}}_{{\hat{\beta }}_{j}}$ to assess correlation direction and significance. Positive ${\hat{\beta }}_{j}$ values indicate positive correlation with the neuropsychological measure, negative values indicate inverse correlation, and zero indicates no correlation. *p*-values were calculated using a *t*-distribution with 24 degrees of freedom to test the null hypothesis of no association. Given that 29,603 lncRNAs were tested against each neuropsychological variable, *p*-values were initially adjusted for multiple comparisons using both Bonferroni^57^ and FDR.^58–60^ However, due to the high risk of instability associated with our sample size, only the more conservative Bonferroni-corrected *p*-values are reported to ensure robust findings.^57–60^

### Predictive power of lncRNAs in AD

The Predictive Power Score (PPS) measures predictive relationships between variables, overcoming limitations of traditional correlation by capturing non-linear, categorical, and asymmetric associations.^61^ In our case, it evaluates the ability of lncRNA expression to predict neuropsychological performance scores across the entire range of the data, capturing non-linear relationships that a simple group-to-group shift would not satisfy. Unlike correlation analysis and GLMs, PPS identifies directional predictive strength by evaluating a Decision Tree model's out-of-sample performance against naive baselines. Here we used the PPS as implemented in the ppsr^62^ package of R to quantify the prediction ability of lncRNA*_j_* (*j* = 1, 2, …,29809) on the neuropsychological variable *y_i_* (*i* *=* 1, 2, 3,…,25).

### Prioritization of lncRNAs

To address the potential pleiotropic effects of lncRNAs on AD neuropsychological architecture, we prioritized their roles using four complementary analytical approaches.

First, we employed Multivariate Analysis of Variance (MANOVA) to assess the joint association between lncRNAs expression and neuropsychological profiles. In this case, the dependent variables correspond to the 25 neuropsychological variables (Table 2) and the predictors to each of the 29,809 lncRNAs. All neuropsychological variables were standardized using the scale() function from R to mitigate scale heterogeneity. The multivariate linear model was specified as **Y** ∼ lncRNA*_j_* + AD + Age + Sex + Schooling, where **Y** ∈ ℝ^30^ ^×^ ^25^ represents the standardized neuropsychological assessment matrix for 30 participants. Subsequently, the associated *p*-value for the *j*-th lncRNA was extracted from the fitted model and only lncRNAs with *p*_MANOVA_ < 0.01 were selected for further analysis.

In our second approach, Bonferroni-corrected *p*-values from correlation analyses (see above) were used to generate a binary matrix **M** ∈ {0,1}^9850^ ^×^ ^25^, indicating whether each lncRNA was significantly associated with each neuropsychological variable at the 5% significance level. Pairwise Hamming distances between the 9850 AD-associated lncRNAs were then computed to construct the distance matrix **D** ∈ ℝ^9850^ ^×^ ^9850^. Multidimensional scaling^63^ was applied to embed the lncRNAs in a 3D space, from which Mahalanobis distances were calculated.^64^ lncRNAs with Mahalanobis distances exceeding the adjusted χ^2^_3,1−0.05/(25_ _×_ _9850)_ = 33.95 critical threshold were prioritized.

Thirdly, we applied the transformation *f*(*x*)=-log_10_(*x*) on the Bonferroni-corrected *p*-value derived from the correlation analysis. Only lncRNAs with effects on at least one neuropsychological variable were included. The resulting values were scaled using the scale() function in R, obtaining a matrix **P** ∈ ℝ^20523^ ^×^ ^12^. Subsequently, Mahalanobis distances were calculated on this matrix and lncRNAs exceeding the χ^2^_3,1−0.05/(25×20523)_ = 56.45 critical threshold were prioritized.

Finally, we utilized PPS to filter lncRNAs, retaining only those with PPS values greater than 0.3. Mahalanobis distances were then calculated on the resulting matrix **W** ∈ ℝ^160^ ^×^ ^25^, where rows correspond to the selected lncRNAs and columns to neuropsychological variables. lncRNAs with Mahalanobis distances exceeding the critical threshold χ^2^_25,0.05_ = 37.65 were prioritized.

## Results

### lncRNA signatures contributing to neuropsychological manifestations of AD

We collected data from 30 individuals (22 [73.3%] females, 15 [50%] with AD) through our clinical evaluation protocols. Table 1 summarizes the demographic and clinical characteristics of all participants.

**Table 1.** Demographic and clinical characteristics of individuals included in this study.

Variable	Cases	Controls	*p ^a^*
	(*n* = 15)	(*n* = 15)	
	**Mean (SD) [Median]**		
Age (years)	77.5 (8.5) [76]	82.1 (8.6) [82]	0.261
Age of onset (years)	72.9 (7.5) [71]	-	*-*
*AVMR*			
Yes	9.9 (4.4) [11]	11.7 (4.1) [14]	0.127
No	8.6 (5.2) [8]	11.9 (5.2) [15]	0.070
*BNT*			
Spontaneous	16.2 (10.4) [13]	37.5 (13.9) [42]	<0.001
Semantic	1.6 (0.7) [1]	2.2 (1) [2]	0.075
Total	16.9 (10.8) [14]	38.7 (14.2) [42]	<0.001
FAST	3.7 (0.5) [4]	2.5 (0.6) [2]	<0.001
Lawton & Brody	1.7 (1.4) [2]	0.3 (0.8) [0]	0.003
MMSE	13.9 (9.5) [17]	25.2 (5.6) [27]	0.001
MoCA	5.5 (5.3) [4]	25.9 (2.9) [26]	<0.001
Phonological fluency			
Letter “a”	3.8 (2.6) [3]	8.7 (4.6) [8]	0.004
Letter “f”	4.7 (3.1) [4]	8.6 (5.2) [7]	0.032
Letter “s”	3.6 (2.3) [3]	8.7 (4.8) [8]	0.002
Verbal fluency			
Letter “a”	4.3 (2.3) [4]	11.2 (3.7) [12]	<0.001
Letter “c”	5.9 (2.6) [6]	9.3 (3.2) [10]	0.004
*ROCFT*			
Copy	10.6 (9.4) [8]	26.5 (11.6) [31]	0.002
Recall	3.5 (2.1) [3]	8.1 (4.7) [9]	0.003
*Stroop test*			
Colors	28.3 (12.2) [25]	42.8 (18.4) [38]	0.031
Words	44.4 (13.4) [42]	68.6 (20.2) [67]	0.002
*TMT*			
Part A	170.1 (47.4) [179]	122.1 (74.8) [98]	0.029
Part B	302.3 (4) [302]	267.5 (70.5) [300]	0.405
Token test	16.7 (8) [16]	26.7 (9.4) [30]	0.003
*WCST*			
Correct answers	36.1 (22.7) [39]	45.1 (38.5) [32]	0.967
NPE	30.7 (21.6) [29]	23.5 (28.8) [16]	0.074
Perseverant errors	30.1 (16) [28]	22.4 (10.5) [24]	0.152
	**Frequency (%)**	
Sex			1
Female	11 (73.3%)	11 (73.3%)	
Male	4 (26.7%)	4 (26.7%)	
Education level			0.262
None	2 (13.3%)	-	
Elementary	7 (46.7%)	6 (40%)	
High school	-	-	
Tertiary	6 (40%)	9 (60%)	
Postgraduate	-	-	
Marital status			0.753
Not reported	5 (33.3%)	7 (46.7%)	
Married	6 (40%)	5 (33.3%)	
Widowed	4 (26.7%)	3 (20%)	

*^a^* Calculated using a nonparametric Mann-Whitney two-sided test. AVMR: Auditory-verbal memory recognition; BNT: Boston Naming Test; FAST: Functional Assessment Screening Tool; MMSE: Mini-Mental State Examination; MoCA: Montreal Cognitive Assessment; ROCFT: Rey–Osterrieth Complex Figure Test; TMT: Trail Making Test; WCST: Wisconsin Card Sorting Test.

Figure 1 depicts the Manhattan plot for those neuropsychological variables with statistically significant results after correcting for multiple testing using Bonferroni's method. We identified lncRNAs whose expression correlated with performance in AD across 12/25 (48%) neuropsychological variables (Table 2). Correlation analysis of the BNT subtests identified 195 long lncRNAs potentially associated with the Number of Spontaneous Clues and 586 lncRNAs correlated with the Total Number of Correct answers (Supplemental Table 1). Among these, lncRNAs located within the *EPHB1*, *DLEU1*, and *CHRNA4* genes showed positive correlations with the Number of Spontaneous Clues, whereas the expression of ENST00000565094 (*HEATR4*) was negatively correlated (Table 2). Similarly, lncRNAs mapping to *EPHB1*, *CHRNA4*, and *CEBPB* exhibited positive correlations with the Total Number of Correct answers. In contrast, increased expression of ENST00000565094 (*HEATR4*) was associated with a decrease in this BNT subtest score (Table 2).

**Figure 1.**
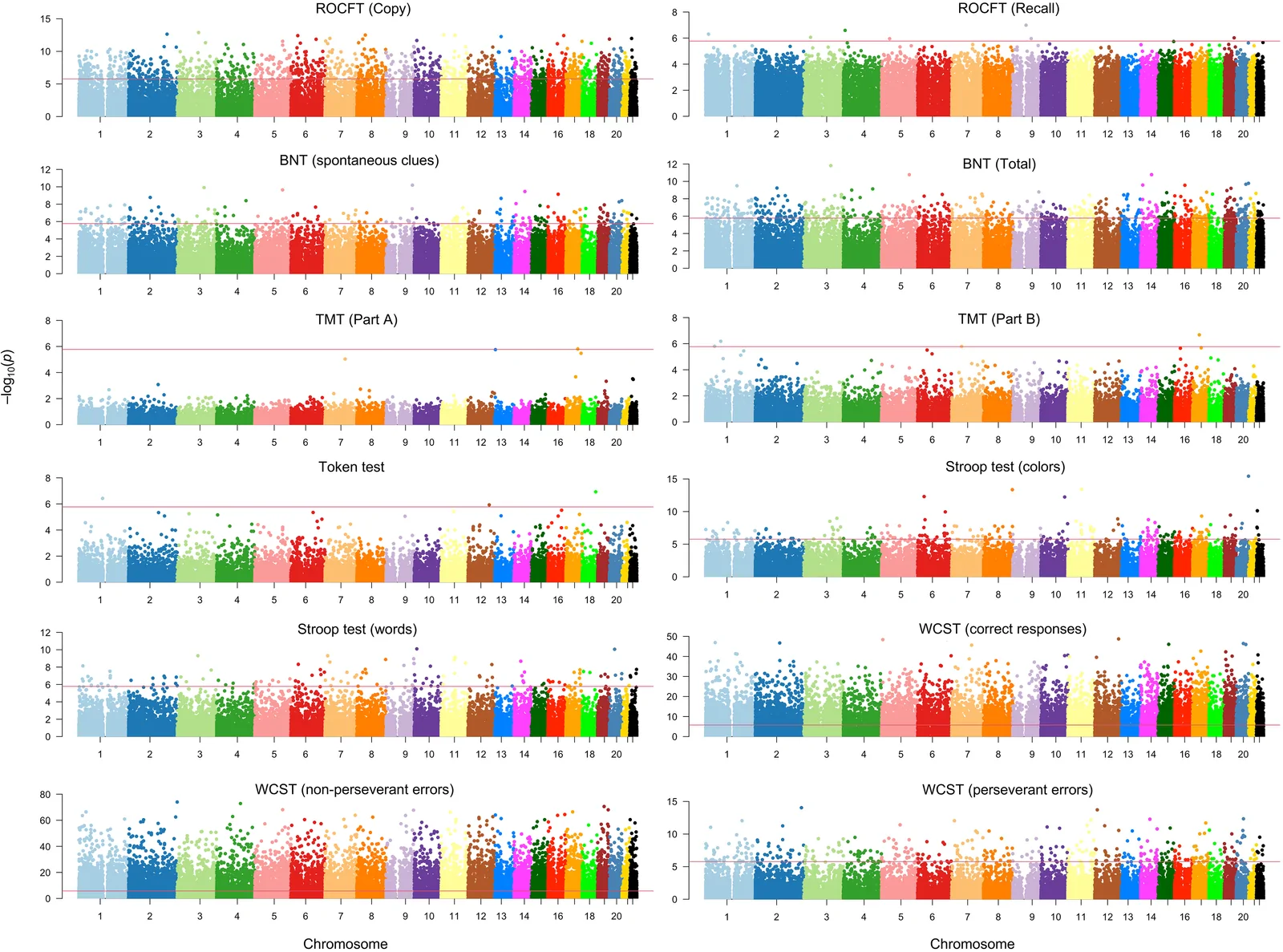
Manhattan plots showing lncRNA signatures correlated with neuropsychological variables in a sample of individuals with AD and healthy controls from Barranquilla, Colombia. The horizontal line corresponds to the Bonferroni's threshold.

**Table 2.** Top lncRNAs correlated with AD by neuropsychological variable.

Neuropsychological test	Transcript	Chr	Position*^a^*	Closest gene*^b^*	Biotype	$\hat{\beta }\left({\hat{\text{SE}}}_{\hat{\beta }}\right)$	*p*_Bonferroni_
*BNT*							
Spontaneous	T367347	9	131,975,444	*RP11-247A12.2*	Antisense	0.683 (0.105)	1.92 × 10^−6^
	ENST00000488154	3	134,514,474	*EPHB1*	Protein coding	0.606 (0.094)	3.62 × 10^−6^
	ENST00000606683	5	139,024,045	*CTD-3224K15.3*	lincRNA	0.432 (0.068)	6.81 × 10^−6^
	ENST00000565094	14	73,945,189	*HEATR4*	Protein coding	−1.062 (0.169)	1.04 × 10^−5^
	TCONS_00024398	16	51,558,114	*HNRNPA1P48*	pseudogene	1.278 (0.207)	2.15 × 10^−5^
	ENST00000413708	2	107,978,775	*AC006227.1*	lincRNA	0.599 (0.099)	5.03 × 10^−5^
	ENST00000486895	13	50,656,307	*DLEU1*	Protein coding	0.648 (0.108)	6.47 × 10^−5^
	ENST00000370257	20	61,991,340	*CHRNA4*	Protein coding	0.627 (0.107)	1.17 × 10^−4^
	ENST00000503593	4	146,998,998	*RP11-203B7.1*	lincRNA	−0.561 (0.095)	1.18 × 10^−4^
	NR_125739	20	48,801,140	*CEBPB*	Protein coding	0.496 (0.085)	1.61 × 10^−4^
Total	ENST00000488154	3	134,514,474	*EPHB1*	Protein coding	0.651 (0.092)	4.62 × 10^−8^
	T367347	9	131,975,444	*RP11-247A12.2*	Antisense	0.721 (0.102)	5.35 × 10^−8^
	ENST00000565094	14	73,945,189	*HEATR4*	Protein coding	−1.123 (0.167)	4.92 × 10^−7^
	ENST00000606683	5	139,024,045	*CTD-3224K15.3*	lincRNA	0.452 (0.067)	5.01 × 10^−7^
	ENST00000370257	20	61,991,340	*CHRNA4*	Protein coding	0.664 (0.104)	4.93 × 10^−6^
	NR_125739	20	48,801,140	*CEBPB*	Protein coding	0.53 (0.083)	6.37 × 10^−6^
	TCONS_00022695	14	29,530,332	*RP11-148E17.1*	lincRNA	−0.529 (0.084)	7.91 × 10^−6^
	TCONS_00024398	16	51,558,114	*HNRNPA1P48*	pseudogene	1.282 (0.203)	8.36 × 10^−6^
	ENST00000606343	1	156,607,575	*RP11-284F21.9*	lincRNA	0.429 (0.068)	9.47 × 10^−6^
	ENST00000413708	2	107,978,775	*AC006227.1*	lincRNA	0.606 (0.098)	1.75 × 10^−5^
*ROCFT*							
Copy	ENST00000427391	X	130,836,680	*FIRRE*	lincRNA	1.132 (0.147)	4.76 × 10^−10^
	ENST00000595232	3	106,991,514	*LINC00883*	lincRNA	1.118 (0.151)	3.90 × 10^−9^
	ENST00000419640	2	192,093,891	*AC092614.2*	lincRNA	−0.415 (0.057)	6.82 × 10^−9^
	ENST00000528792	11	12,870,342	*TEAD1*	Protein coding	0.801 (0.11)	8.77 × 10^−9^
	T343001	8	42,260,903	*VDAC3*	Protein coding	0.581 (0.08)	9.17 × 10^−9^
	ENST00000512200	11	68,638,132	*RP11-757G1.6*	lincRNA	−0.525 (0.072)	9.74 × 10^−9^
	ENST00000531347	11	69,918,540	*ANO1-AS2*	Antisense	1.712 (0.235)	9.96 × 10^−9^
	ENST00000569677	16	79,246,608	*RP11-679B19.2*	lincRNA	−0.899 (0.124)	1.12 × 10^−8^
	MICT00000302088	6	33,945,117	*MIR1275*	miRNA	−0.671 (0.092)	1.15 × 10^−8^
	ENST00000476738	13	50,656,307	*DLEU1*	Protein coding	1.096 (0.152)	1.60 × 10^−8^
Recall	ENST00000438699	9	66,523,532	*RP11-262H14.3*	lincRNA	−1.378 (0.259)	3.05 × 10^−3^
	T260896	4	8,645,137	*CPZ*	Protein coding	−0.757 (0.147)	7.62 × 10^−3^
	T003446	1	14,152,137	*PRDM2*	Protein coding	1.335 (0.266)	1.49 × 10^−2^
	ENCT00000301655	3	33,316,741	*FBXL2*	Protein coding	−0.835 (0.17)	2.60 × 10^−2^
	ENST00000596497	19	48,968,865	*KCNJ14*	Protein coding	−0.918 (0.187)	2.85 × 10^−2^
	ENST00000491609	X	134,022,984	*MOSPD1*	Protein coding	0.991 (0.203)	3.11 × 10^−2^
	TCONS_00016056	9	91,825,614	*SHC3*	Protein coding	−0.431 (0.088)	3.25 × 10^−2^
	ENST00000649444	5	40,463,664	*AC108105.1*	miRNA	−0.985 (0.202)	3.32 × 10^−2^
*Stroop test*							
Colors	TCONS_00028265	20	62,030,713	*KCNQ2*	Protein coding	0.564 (0.069)	1.12 × 10^−11^
	ENST00000512200	11	68,638,132	*RP11-757G1.6*	lincRNA	−0.417 (0.055)	1.20 × 10^−9^
	ENST00000502167	8	144,089,170	*LY6E-DT*	lincRNA	−0.757 (0.1)	1.33 × 10^−9^
	ENST00000489721	6	32,135,320	*PPT2-EGFL8*	Protein coding	0.536 (0.074)	1.49 × 10^−8^
	ENST00000440007	10	119,243,806	*EMX2OS*	lincRNA	0.481 (0.067)	1.74 × 10^−8^
	ENST00000577456	22	20,632,023	*AC011718.2*	lincRNA	−0.262 (0.04)	2.24 × 10^−6^
	ENST00000434437	6	138,266,503	*RP11-10J5.1*	lincRNA	−0.686 (0.106)	3.35 × 10^−6^
	T172585	19	30,265,492	*CCNE1*	Protein coding	−0.247 (0.039)	1.03 × 10^−5^
	ENST00000572864	17	45,148,812	*RP11-156P1.3*	Processed transcript	0.634 (0.102)	1.48 × 10^−5^
	NR_125764	3	164,924,748	*RP11-85M11.2*	lincRNA	0.325 (0.053)	3.25 × 10^−5^
Words	ENST00000448272	10	13,457,202	*RP11-445K13.2*	Antisense	−0.322 (0.049)	2.37 × 10^−6^
	ENST00000601901	20	26,168,268	*MIR663A*	Processed transcript	−0.375 (0.058)	2.63 × 10^−6^
	ENCT00000408932	7	12,725,221	*CTD-2320J21.1*	pseudogene	−0.311 (0.05)	1.45 × 10^−5^
	T247315	3	103,841,735	*RP11-40M23.1*	pseudogene	−0.26 (0.042)	1.46 × 10^−5^
	ENST00000512200	11	68,638,132	*RP11-757G1.6*	lincRNA	−0.265 (0.043)	2.32 × 10^−5^
	ENST00000603624	9	138,354,573	*PPP1R26-AS1*	Antisense	0.257 (0.042)	3.24 × 10^−5^
	ENST00000502167	8	144,089,170	*LY6E-DT*	lincRNA	−0.479 (0.079)	3.99 × 10^−5^
	ENST00000616315	11	65,193,022	*NEAT1*	lincRNA	−0.335 (0.055)	4.42 × 10^−5^
	ENST00000649005	14	53,258,503	*GNPNAT1*	Protein coding	0.226 (0.038)	6.24 × 10^−5^
	ENST00000454029	7	23,719,814	*FAM221A*	Protein coding	0.288 (0.048)	7.81 × 10^−5^
*TMT*							
Part A	ENST00000585495	17	59,460,762	*BCAS3*	Protein coding	−38.717 (6.125)	4.63 × 10^−2^
Part B	ENST00000590478	17	33,558,469	*RP11-799D4.4*	Antisense	−64.015 (8.934)	6.25 × 10^−3^
	ENST00000446238	1	75,593,515	*RP11-510C10.2*	Antisense	122.146 (18.279)	1.95 × 10^−2^
	ENST00000445226	1	44,410,044	*RP11-7O11.3*	Antisense	−76.174 (12.064)	4.71 × 10^−2^
	T323724	7	49,168,842	*AC010971.1*	lincRNA	141.189 (22.41)	4.88 × 10^−2^
Token test	GSE61474_TCONS_00156017	18	68,132,153	*RPS2P6*	pseudogene	0.744 (0.141)	3.53 × 10^−3^
	TCONS_l2_00001470	1	118,359,347	*AL390877.1*	pseudogene	−1.041 (0.205)	1.12 × 10^−2^
	TCONS_00020904	12	105,836,748	*C12orf75*	Protein coding	0.262 (0.054)	3.52 × 10^−2^
*WCST*							
Correct answers	ENST00000543558	12	119,083,120	*RP11-121J20.1*	lincRNA	−0.994 (0.067)	5.74 × 10^−45^
	ENST00000505626	5	6,582,251	*LINC01018*	lincRNA	0.813 (0.055)	1.23 × 10^−44^
	ENST00000592775	6	146,136,434	*RP11-545I5.3*	Antisense	0.714 (0.048)	1.52 × 10^−44^
	ENST00000445551	1	47,897,805	*FOXD2-AS1*	Antisense	−0.677 (0.047)	3.71 × 10^−43^
	T198116	2	121,876,102	*AC067960.1*	lincRNA	−0.803 (0.056)	5.72 × 10^−43^
	ENST00000373508	20	36,305,312	*RP4-640H8.2*	lincRNA	0.435 (0.03)	1.07 × 10^−42^
	T119156	15	72,891,278	*RP11-361M10.3*	pseudogene	0.778 (0.054)	2.64 × 10^−42^
	HSALNT0289277	20	48,657,243	*TRERNA1*	lincRNA	1.016 (0.071)	3.17 × 10^−42^
	ENST00000482799	7	98,628,218	*SMURF1*	Protein coding	1.11 (0.078)	7.03 × 10^−42^
	ENST00000463529	X	48,755,793	*PQBP1*	Protein coding	−0.79 (0.056)	5.92 × 10^−41^
NPE	ENST00000426962	2	242,913,429	*AC131097.3*	Antisense	0.929 (0.051)	3.09 × 10^−70^
	ENST00000602483	4	119,199,910	*SNHG8*	lincRNA	1.29 (0.071)	4.32 × 10^−69^
	ENST00000593211	19	32,413,790	*CTC-360P9.5*	lincRNA	−0.733 (0.041)	8.23 × 10^−67^
	ENST00000521563	5	139,483,425	*MALINC1*	Antisense	1.22 (0.07)	2.29 × 10^−64^
	NR_125345	19	52,892,093	*ZNF528-AS1*	Antisense	−0.861 (0.049)	2.84 × 10^−64^
	TCONS_00015892	9	137,419,027	*RP11-473E2.4*	lincRNA	1.121 (0.064)	5.90 × 10^−64^
	ENST00000638682	17	33,895,128	*RP11-1094M14.11*	lincRNA	1.175 (0.068)	1.11 × 10^−62^
	T007806	1	35,497,914	*ZMYM6*	Protein coding	0.96 (0.055)	1.26 × 10^−62^
	ENST00000532068	11	45,755,545	*CTD-2210P24.1*	lincRNA	1.035 (0.06)	1.87 × 10^−62^
	HBMT00000551071	16	84,651,102	*COTL1*	Protein coding	0.957 (0.056)	1.13 × 10^−60^
Perseverant errors	T209245	2	230,613,416	*TRIP12*	Protein coding	0.651 (0.084)	2.71 × 10^−10^
	ENCT00000088265	12	12,420,309	*LRP6*	Protein coding	0.778 (0.102)	5.68 × 10^−10^
	ENST00000479646	20	36,888,608	*KIAA1755*	Protein coding	0.393 (0.054)	1.35 × 10^−8^
	FTMT25600002825	14	65,347,312	*SPTB*	Protein coding	−0.591 (0.082)	1.57 × 10^−8^
	TCONS_00019769	11	115,754,859	*AP000797.3*	lincRNA	0.821 (0.114)	2.14 × 10^−8^
	ENCT00000408932	7	12,725,221	*CTD-2320J21.1*	pseudogene	0.485 (0.068)	2.65 × 10^−8^
	ENST00000608183	1	182,098,445	*GS1-122H1.2*	lincRNA	0.592 (0.083)	2.69 × 10^−8^
	T152907	17	65,673,974	*PITPNC1*	Protein coding	0.433 (0.061)	5.91 × 10^−8^
	T286073	5	92,934,175	*NR2F1*	Protein coding	−0.473 (0.068)	1.14 × 10^−7^
	ENST00000511243	11	96,180,296	*JRKL-AS1*	Protein coding	−0.821 (0.119)	1.41 × 10^−7^

*^a^* UCSC GRCh37/hg19 coordinates. ^*b*^ Retrieved using the annotate_with_nearest_gene() function from the topr^65^ package in R. $\hat{\beta }$ : regression coefficient; Chr: chromosome; *p*_Bonferroni:_ Bonferroni-corrected *p*-value; ${\hat{SE}}_{\hat{\beta }}$ : estimated standard error of $\hat{\beta }$ . Conventions as in Table 1.

A total of 1310 lncRNAs were significantly correlated with performance of the components of the Rey-Osterrieth Complex Figure Test (ROCFT) (Supplemental Table 2). Several of these transcripts were either positively or negatively associated with scores on the Copy and Recall components of the ROCFT and mapped to multiple protein-coding genes, including *TEAD1*, *VDAC3*, *DLEU1*, *CPZ*, *PRDM2*, *FBXL2*, and *SHC3* (Table 2). Elevated expression of T343001 (*VDAC3*) was associated with improved performance on the Copy component, whereas higher expression of ENCT00000301655 (*FBXL2*) correlated with poorer scores on the Recall component (Table 2).

Correlation analyses between lncRNA expression levels and performance on the Colours component of the Stroop test identified 184 transcripts (Supplemental Table 3). The strongest positive correlations with improved performance were observed for lncRNAs mapping to the *KCNQ2* and *PPT2-EGFL8* genes, as well as the long intergenic non-coding RNA (lincRNA) ENST00000440007 (*EMX2OS*) (Table 2). In contrast, lncRNAs mapping to the *CCNE1* gene and the *RP11-10J5.1* lincRNA exhibited significant negative correlations with Colors test performance (Table 2).

Additionally, 134 lncRNAs were significantly correlated with the Number of Words component of the Stroop test following multiple testing correction (Supplemental Table 4). Specifically, lncRNAs mapping to *LY6E-DT* and *NEAT1* were negatively correlated with performance, whereas lncRNAs within the *GNPNAT1* and *FAM221A* genes were associated with poorer outcomes (Table 2).

Analysis of the association between lncRNA expression and performance on Parts A and B of the TMT identified five transcripts with significant correlations (Table 2). Specifically, ENST00000585495 (*BCAS3*), ENST00000590478 (*RP11-799D4.4*), and ENST00000445226 (*RP11-7O11.3*) were associated with faster completion times on the TMT. In contrast, ENST00000446238 (*RP11-510C10.2*) and T323724 (*AC010971.1*) correlated with longer completion times (Table 2).

Evaluation of the potential association between the Token test and lncRNA expression identified three transcripts (Table 2). Of these, lncRNAs mapping to *RPS2P6* and *C12orf75* were associated with better scores on the test, whereas the expression in the TCONS_l2_00001470 (*AL390877.1*) is statistically significantly associated with poorer performance (Table 2).

Finally, we identified several transcripts significantly correlated with performance metrics of the Wisconsin Card Sorting Test (WCST), including the number of correct responses, non-perseverative errors (NPE), and perseverative errors, after correction for multiple testing (Supplemental Table 4). Table 2 presents the top 10 lncRNAs associated with each WCST subtest. Notably, lncRNAs mapping to *TRERNA1* and *SMURF1* were positively correlated with a higher number of correct responses, while expression of *ZMYM6* was positively correlated with an increased number of non-perseverative errors. Additionally, the expression of ENST00000511243 (*JRKL-AS1*) was negatively correlated with the NPE (Table 2).

### Prioritization of lncRNAs based on statistical significance

Table 3 summarizes the results of the MANOVA-based priorization method for identifying lncRNAs statistically significantly associated to AD neuropsychological architecture. We prioritized 33 lncRNAs when a Type I error of 1% was used. Of these, 16 (48.5%) lcRNAs mapped to protein coding genes, 10 (30.3%) to lincRNAs, 5 (15.2%) to Antisense genes, 1 (3%) to a polymorphic pseudogene and 1 (3%) to a processed transcript.

**Table 3.** Prioritized lncRNAs associated to ad neuropsychological architecture based on MANOVA.

Transcript ID	Chr	Position	*p*	Closest gene	Biotype
ENCT00000200087	19	913,278	2.04 × 10^−3^	*R3HDM4*	Protein coding
ENST00000513259	5	138,609,793	3.14 × 10^−3^	*SIL1*	Protein coding
ENST00000593790	10	75,256,049	3.45 × 10^−3^	*RP11-137L10.6*	Processed transcript
ENST00000439529	20	11,890,052	3.54 × 10^−3^	*BTBD3*	Protein coding
NR_026690	7	73,149,398	3.90 × 10^−3^	*LINC00035*	Antisense
ENST00000511805	4	3,319,766	4.14 × 10^−3^	*RGS12*	Protein coding
ENST00000442606	1	216,252,415	4.31 × 10^−3^	*USH2A*	Protein coding
ENST00000523792	8	122,966,879	5.46 × 10^−3^	*RP11-398G24.2*	lincRNA
MICT00000183281	2	2,612,835	5.60 × 10^−3^	*AC018685.1*	lincRNA
ENST00000502300	17	48,580,773	6.05 × 10^−3^	*RP11-94C24.6*	lincRNA
ENST00000593246	13	31,478,894	6.29 × 10^−3^	*TEX26-AS1*	Antisense
ENST00000515194	4	2,937,273	6.58 × 10^−3^	*NOP14-AS1*	Antisense
ENST00000596289	4	56,233,121	6.62 × 10^−3^	*SRD5A3*	Protein coding
T109592	14	95,535,772	6.83 × 10^−3^	*DICER1*	Protein coding
ENST00000642131	1	11,683,615	6.86 × 10^−3^	*RP1-69M21.2*	lincRNA
ENST00000414354	3	196,730,621	7.05 × 10^−3^	*MFI2*	Protein coding
ENST00000526041	11	108,873,192	7.44 × 10^−3^	*RP11-25I9.2*	lincRNA
ENST00000648003	14	64,980,670	7.59 × 10^−3^	*ZBTB1*	Protein coding
ENST00000472110	22	42,084,943	7.86 × 10^−3^	*NHP2L1*	Protein coding
NR_040288	1	19,592,475	8.33 × 10^−3^	*AKR7L*	Polymorphic pseudogene
ENST00000534123	9	100,013,111	8.44 × 10^−3^	*CCDC180*	Protein coding
ENST00000584262	17	57,508,230	8.89 × 10^−3^	*RP11-567L7.6*	lincRNA
TCONS_00004319	2	81,152,585	9.06 × 10^−3^	*AC084193.1*	lincRNA
ENST00000424689	1	8,278,250	9.24 × 10^−3^	*RP11-431K24.4*	lincRNA
ENST00000582092	18	23,603,577	9.25 × 10^−3^	*SS18*	Protein coding
ENST00000583492	17	79,886,277	9.30 × 10^−3^	*MAFG-AS1*	Antisense
ENST00000590491	19	2,610,153	9.44 × 10^−3^	*GNG7*	Protein coding
ENST00000592651	17	75,543,022	9.49 × 10^−3^	*RP11-13K12.1*	lincRNA
ENST00000530249	11	29,735,456	9.60 × 10^−3^	*CTD-3138F19.1*	lincRNA
ENST00000527261	11	10,329,860	9.65 × 10^−3^	*AMPD3*	Protein coding
ENST00000506902	5	60,457,897	9.94 × 10^−3^	*SMIM15*	Protein coding
ENST00000438431	10	13,570,516	9.96 × 10^−3^	*BEND7*	Protein coding
ENST00000645215	8	128,334,450	1.00 × 10^−2^	*CASC8*	Antisense

We identified a total of 57 transcripts statistically associated with at least two neuropsychological variables using our second prioritization approach. Among these, 18 transcripts (31.6%) were correlated with two variables, three transcripts (5.3%) with three variables, 26 transcripts (45.6%) with four variables, 9 transcripts (15.8%) with five variables, and 1 (1.8%) transcript with six variables out of the 25 tested (Supplemental Table 5). Table 4 highlights those lncRNAs statistically significantly correlated with five or more neuropsychological variables.

**Table 4.** Prioritization of lncRNAs based on the hamming distance. Conventions as in Table 2.

Transcript ID	Chr	Position	MD	Closest gene	Biotype	Total tests
FTMT27800001222	20	30,326,666	71.6	*TPX2*	Protein coding	6
ENCT00000408932	7	12,725,221	110.0	*CTD-2320J21.1*	Pseudogene	5
T247315	3	103,841,735	86.8	*RP11-40M23.1*	Pseudogene	5
ENST00000448272	10	13,457,202	80.1	*RP11-445K13.2*	Antisense	5
ENST00000649005	14	53,258,503	77.5	*GNPNAT1*	Protein coding	5
ENST00000616315	11	65,193,022	71.6	*NEAT1*	lincRNA	5
T315255	6	167,372,390	71.5	*RNASET2*	Protein coding	5
ENST00000443642	1	155,287,737	68.8	*FDPS*	Protein coding	5
ENST00000534297	11	121,962,253	60.4	*RP11-166D19.1*	Sense overlapping	5
ENST00000562166	16	2,647,882	38.2	*PDPK1*	Protein coding	5

MD: Mahalanobis distance.

Using our *p*-value-based prioritization framework for lncRNAs, we identified 171 transcripts statistically significantly correlated to our neuropsychological variables (Supplemental Table 6). Among these, 52 (30.4%) transcripts correlated with one neuropsychological variable, 53 (31.0%) with two variables, 33 (19.3%) with three variables, 20 (11.7%) with four variables, 12 (7.0%) with five variables, and 1 (0.6%) with six variables, out of the 25 analyzed. Table 5 lists the lncRNAs correlated with five or more neuropsychological variables.

**Table 5.** Prioritization of lncRNAs based on the *p-*value transformation. Conventions as in Table 3.

Transcript ID	Chr	Position	MD	Closest gene	Biotype	Total tests
FTMT27800001222	20	30,326,666	71.6	*TPX2*	Protein coding	6
ENCT00000408932	7	12,725,221	110.0	*CTD-2320J21.1*	Pseudogene	5
ENST00000440007	10	119,243,806	156.2	*CTA-109P11.4*	lincRNA	5
ENST00000443642	1	155,287,737	68.8	*FDPS*	Protein coding	5
ENST00000448272	10	13,457,202	80.1	*RP11-445K13.2*	Antisense	5
ENST00000454029	7	23,719,814	58.8	*FAM221A*	Protein coding	5
ENST00000534297	11	121,962,253	60.4	*RP11-166D19.1*	Sense overlapping	5
ENST00000601901	20	26,168,268	83.6	*MIR663A*	Processed transcript	5
ENST00000603624	9	138,354,573	62.9	*PPP1R26-AS1*	Antisense	5
ENST00000616315	11	65,193,022	71.6	*NEAT1*	lincRNA	5
ENST00000649005	14	53,258,503	77.5	*GNPNAT1*	Protein coding	5
T247315	3	103,841,735	86.8	*RP11-40M23.1*	Pseudogene	5
T315255	6	167,372,390	71.5	*RNASET2*	Protein coding	5

### PPS by neuropsychological variables

Figure 2 shows the distribution of the PPS of lncRNAs across neuropsychological variables. On average, 21.1% of lncRNAs have a negligible PPS; the minimum value is 10.12% (Recall in the ROCFT) and the maximum is 43.97% (Semantic Clues in BNT).

**Figure 2.**
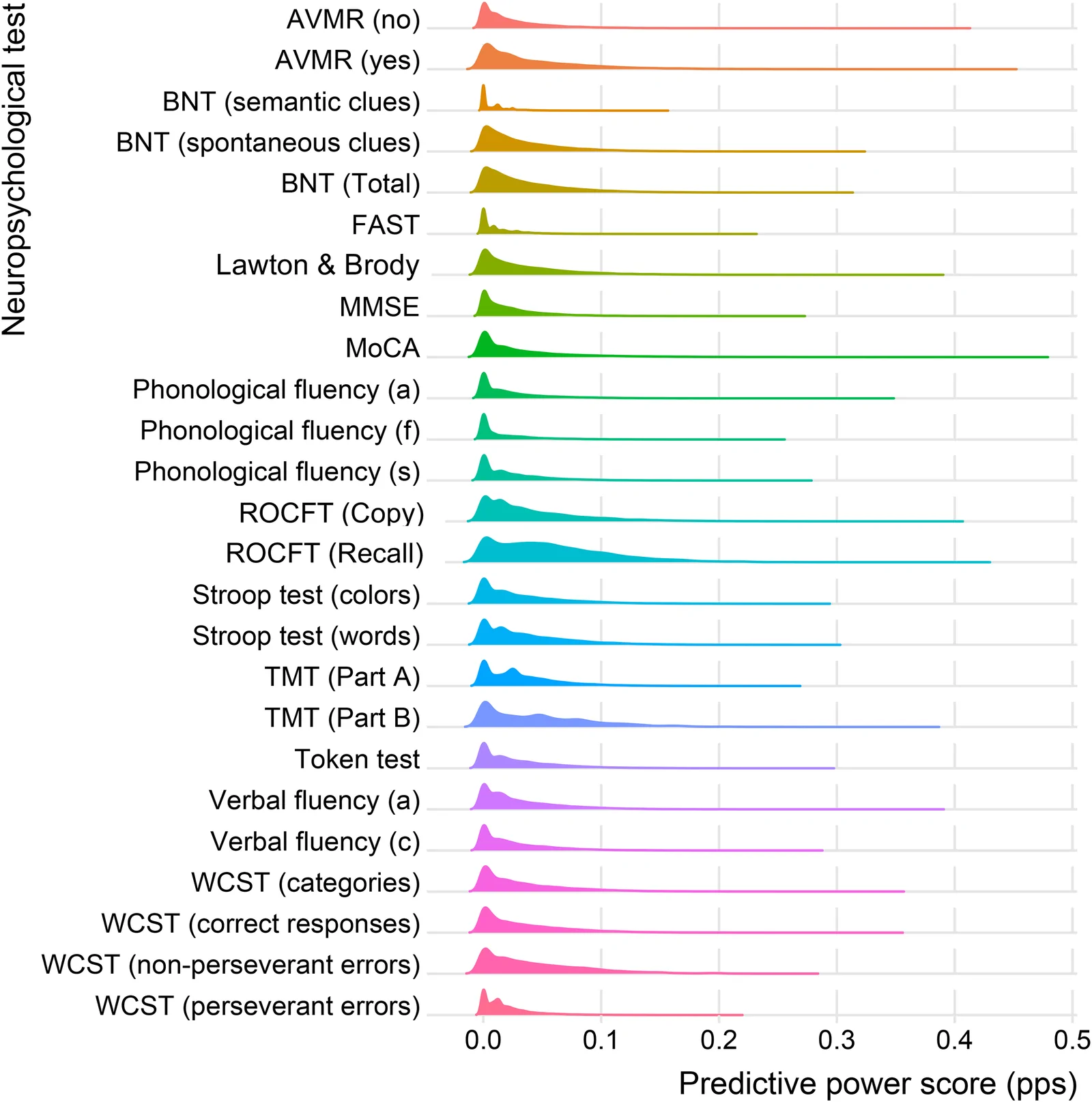
PPS distribution based on lncRNA signatures by neuropsychological test. Conventions as in Table 1.

Table 6 presents the top five lncRNAs according to the PPS. Several of these transcripts are located within genes implicated in biological processes that are commonly disrupted in individuals with AD, such as synaptic function, neuroinflammation, and amyloid processing. Across all neuropsychological variables, the lcRNA with the maximum PPS was TCONS_00023350 (*XLOC_011212*; PPS = 0.467), followed by ENST00000547709 (*C17orf49*; PPS = 0.444), ENST00000418612 (*LINC01219*; PPS = 0.440), all of them in the MoCA test. Other identified transcripts are harbored in genes associated to the pathophysiological changes typically observed in AD (i.e., *DARS-AS1*, *NREP*, *PGBD1*, *IBA57*, *DOCK7-DT*, *KIRREL3-AS1*, *VPS52* and *FAM117A*) (Table 6).

**Table 6.** Top 5 lncRNAs with the highest PPS by neuropsychological test. Conventions as in Table 1.

Variable	Transcript	Chr	Position	Closest gene	PPS
AVMR (no)	ENST00000598565	2	136,757,983	*DARS-AS1*	0.405
	ENST00000619671	19	9,649,415	*ZNF426-DT*	0.286
	ENST00000565094	14	73,945,189	*HEATR4*	0.280
	TCONS_00013496	7	73,668,848	*RFC2*	0.269
	ENST00000422010	9	85,066,571	*RASEF*	0.258
AVMR (yes)	T287304	5	111,047,834	*NREP*	0.439
	ENST00000624792	22	27,439,963	*MIATNB*	0.390
	ENST00000498091	6	107,019,850	*RTN4IP1*	0.388
	ENST00000426702	3	23,845,515	*UBE2E1-AS1*	0.376
	TCONS_l2_00024682	6	28,281,280	*PGBD1*	0.370
*BNT*					
Spontaneous clues	T032159	1	228,370,283	*IBA57*	0.313
	T299580	6	27,637,229	*LINC01012*	0.307
	ENST00000554983	14	21,338,438	*AL133371.3*	0.273
	compmerge.9546.pooled.chr1	1	63,169,928	*DOCK7-DT*	0.272
	ENST00000548204	11	126,413,842	*KIRREL3-AS1*	0.269
Semantic clues	ENST00000478934	6	33,218,049	*VPS52*	0.153
	uc001ixf.3	10	34,398,809	*AX765719*	0.128
	FTMT27800001222	20	30,326,666	*TPX2*	0.127
	ENST00000514902	14	103,653,558	*LINC00605*	0.122
	ENST00000458139	1	201,867,228	*AL513217.1*	0.120
Total	T032159	1	228,370,283	*G007274*	0.303
	T299580	6	27,637,229	*G070045*	0.296
	compmerge.9546.pooled.chr1	1	63,169,928	*LINC00466*	0.273
	MICT00000071289	12	798,989	*CATG00000007614.1*	0.273
	ENST00000554983	14	21,338,438	*AL133371.3*	0.270
FAST	ENST00000650187	6	91,030,162	*AL132996.1*	0.227
	T299580	6	27,637,229	*G070045*	0.220
	ENST00000431616	X	102,024,089	*LINC00630*	0.207
	ENST00000529219	11	50,257,789	*AC109635.2*	0.207
	ENST00000608471	2	136,751,455	*DARS-AS1*	0.205
Lawton & Brody	ENST00000576138	17	6,556,783	*C17orf100*	0.379
	ENST00000648691	19	35,581,834	*HPN-AS1*	0.353
	ENST00000585834	2	6,757,540	*MIR7515HG*	0.346
	ENST00000598247	2	40,341,678	*SLC8A1-AS1*	0.340
	ENST00000619671	19	9,649,415	*ZNF426-DT*	0.334
MMSE	ENST00000504382	5	82,236,277	*AC027338.2*	0.265
	ENST00000619671	19	9,649,415	*ZNF426-DT*	0.261
	ENST00000570273	16	56,966,060	*HERPUD1*	0.256
	ENST00000585834	2	6,757,540	*MIR7515HG*	0.241
	ENST00000515519	5	148,442,880	*SH3TC2-DT*	0.237
MoCA	TCONS_00023350	15	36,782,441	*XLOC_011212*	0.467
	ENST00000547709	17	6,919,484	*C17orf49*	0.444
	ENST00000418612	11	2,012,326	*LINC01219*	0.440
	ENST00000589662	18	53,388,549	*AC022031.2*	0.440
	TCONS_00028365	20	30,691,027	*XLOC_013720*	0.437
Phonological fluency					
Letter “a”	T299580	6	27,637,229	*G070045*	0.340
	T032159	1	228,370,283	*G007274*	0.286
	T310585	6	121,725,933	*G072803*	0.256
	ENCT00000423498	8	29,266,216	*CATG00000098361.1*	0.245
	ENST00000514018	17	47,794,949	*FAM117A*	0.244
Letter “f”	ENST00000533964	11	48,035,958	*PTPRJ-AS1*	0.248
	ENST00000514506	17	47,583,019	*AC006487.1*	0.246
	ENCT00000365478	5	174,541,738	*CATG00000082103.1*	0.237
	MICT00000008076	1	36,916,228	*CATG00000112861.1*	0.237
	FTMT27800001222	20	30,326,666	*TPX2*	0.232
Letter “s”	ENCT00000423498	8	29,266,216	*CATG00000098361.1*	0.269
	ENST00000573755	17	6,857,417	*AC027763.2*	0.269
	ENST00000425900	20	7,050,261	*AL096799.1*	0.252
	NR_146917	1	219,086,099	*LINC01710*	0.231
	ENST00000565643	16	84,226,164	*AC009123.1*	0.230
*ROCFT*					
Copy	ENST00000453342	7	124,569,975	*POT1-AS1*	0.394
	ENST00000581360	17	30,822,554	*AC079336.5*	0.377
	ENST00000533670	11	67,654,061	*AP003716.1*	0.359
	ENST00000552284	12	49,392,160	*DDN-AS1*	0.343
	ENST00000595134	19	17,119,999	*AC020908.2*	0.342
Recall	ENST00000484285	9	38,620,731	*FAM201A*	0.413
	ENST00000573737	17	81,168,296	*AC139099.1*	0.323
	ENST00000449694	11	64,188,007	*AP003774.2*	0.314
	ENST00000499194	11	43,930,842	*ALKBH3-AS1*	0.305
	ENST00000462119	6	111,620,231	*REV3L*	0.301
*Stroop test*					
Colors	ENST00000650080	12	69,605,888	*AC127894.1*	0.282
	ENST00000458139	1	201,867,228	*AL513217.1*	0.267
	ENST00000534904	11	114,231,357	*AP002518.1*	0.260
	ENST00000460886	21	47,532,332	*COL6A2*	0.253
	TCONS_00005675	3	182,200,734	*XLOC_002943*	0.246
Words	ENST00000420243	7	39,023,843	*POU6F2-AS2*	0.291
	ENST00000520760	8	675,188	*AC100797.1*	0.256
	ENST00000637043	13	42,999,028	*LINC02341*	0.243
	TCONS_00020953	12	120,718,114	*XLOC_010219*	0.240
	ENST00000456099	22	38,037,839	*Z83844.2*	0.240
*TMT*					
Part A	ENST00000558742	15	73,627,601	*AC068397.2*	0.259
	ENST00000412835	2	175,412,102	*AC010894.3*	0.241
	ENST00000419128	22	37,562,797	*Z82188.2*	0.222
	TCONS_00023778	15	89,897,149	*XLOC_011572*	0.219
	ENST00000421730	7	22,928,990	*FAM126A*	0.213
Part B	ENST00000527286	11	111,789,721	*C11orf52*	0.371
	ENST00000563810	8	41,685,683	*AC027702.1*	0.325
	T109723	14	96,228,074	*G025965*	0.323
	ENST00000494318	8	76,320,149	*HNF4G*	0.321
	ENST00000646635	4	8,321,844	*LINC02517*	0.310
Token test	T299580	6	27,637,229	*G070045*	0.287
	ENST00000609247	1	101,360,484	*AC104506.1*	0.287
	ENST00000619671	19	9,649,415	*ZNF426-DT*	0.282
	ENST00000514018	17	47,794,949	*FAM117A*	0.273
	T310585	6	121,725,933	*G072803*	0.249
Verbal fluency					
Letter “a”	T299580	6	27,637,229	*G070045*	0.380
	ENST00000446167	1	44,410,042	*AL357079.1*	0.282
	ENST00000472650	2	219,136,103	*PNKD*	0.267
	HSALNT0289151	3	8,262,833	*LMCD1-AS1*	0.260
	T044598	10	79,872,357	*G010331*	0.256
Letter “c”	ENCT00000023718	1	31,626,963	*CATG00000053667.1*	0.278
	TCONS_00020963	12	124,688,047	*XLOC_010233*	0.247
	T044598	10	79,872,357	*G010331*	0.241
	T299580	6	27,637,229	*G070045*	0.228
	ENST00000542598	11	73,116,342	*AP000763.3*	0.227
*WCST*					
Categories	ENST00000529069	11	67,653,948	*AP003716.1*	0.345
	ENST00000449694	11	64,188,007	*AP003774.2*	0.333
	ENCT00000094468	12	93,323,279	*CATG00000009962.1*	0.326
	ENST00000533670	11	67,654,061	*AP003716.1*	0.317
	ENST00000637153	5	13,860,412	*AC016576.1*	0.317
Correct answers	ENCT00000459234	9	107,728,382	*CATG00000109323.1*	0.344
	ENST00000513779	5	91,767,910	*AC124854.1*	0.326
	ENST00000501371	12	2,785,167	*CACNA1C-AS1*	0.298
	ENST00000416894	1	150,488,233	*FALEC*	0.287
	ENST00000564808	16	19,125,324	*AC099518.3*	0.282
NPE	ENST00000424371	10	119,232,748	*EMX2OS*	0.270
	ENST00000442103	9	71,437,319	*AL354794.1*	0.268
	T144334	17	25,701,141	*G033592*	0.265
	ENST00000413994	1	241,587,698	*AL359764.1*	0.265
	ENST00000609322	22	37,767,724	*Z94160.2*	0.265
Perseverant errors	ENST00000600308	11	6,223,131	*AC022762.2*	0.214
	T142859	17	16,899,899	*G033260*	0.209
	ENST00000502221	12	130,336,753	*AC055717.1*	0.200
	ENST00000624089	5	140,563,307	*AC244517.4*	0.199
	ENST00000595424	13	52,026,565	*INTS6-AS1*	0.193

### PPS-based prioritization of lncRNAs

We identified 160 lncRNAs (0.54% of the total) with potential predictive power (i.e., PPS > 0.3). Of those, 152 transcripts (95.0%) showed significant predictive power for one neuropsychological variable, 2 (3.75%) for two variables, and 3 (1.25%) for three variables. Table 7 presents the top 18 prioritized lncRNAs (11.25%), several of which map to genes involved in biological processes commonly disrupted in AD, including APP, EEA1, POT1, IBA57, and SLC8A1.

**Table 7.** Prioritized lncRNAs based on the PPS.

Transcript ID	Chr	Position	Closest gene	Biotype	Total
T299580	6	27,637,229	*RP1-15D7.1*	Pseudogene	3
ENCT00000094468	12	93,323,279	*EEA1*	Protein coding	2
T032159	1	228,370,283	*IBA57*	Protein coding	2
ENCT00000459234	9	107,728,382	*RP11-217B7.3*	lincRNA	1
ENST00000453342	7	124,569,975	*POT1*	Protein coding	1
ENST00000458139	1	201,867,228	*LMOD1*	Protein coding	1
ENST00000513779	5	91,767,910	*RP11-348J24.2*	lincRNA	1
ENST00000559008	15	80,665,755	*RP11-210M15.2*	Protein coding	1
ENST00000581360	17	30,822,554	*MYO1D*	Protein coding	1
ENST00000591920	19	28,947,918	*AC005307.3*	lincRNA	1
ENST00000594086	7	156,902,674	*AC006967.1*	Protein coding	1
ENST00000597894	21	27,306,620	*APP*	Protein coding	1
ENST00000598247	2	40,341,678	*SLC8A1*	Protein coding	1
ENST00000598565	2	136,757,983	*AC093391.2*	Antisense	1
ENST00000611423	19	48,111,130	*CTD-2571L23.8*	lincRNA	1
ENST00000619671	19	9,649,415	*ZNF426*	Protein coding	1
NR_125770	13	81,592,526	*RP11-521J24.1*	lincRNA	1
TCONS_00018646	10	132,608,002	*MIR378C*	miRNA	1

## Discussion

In this study, we examined the relationship between lncRNA expression in blood-derived exosomes and domain-specific neuropsychological performance in individuals with AD. Using a comprehensive cognitive battery of tests including the MMSE, MoCA, Functional Assessment Screening Tool, BNT, Verbal and Phonological Fluency, TMT, ROCFT, Stroop test, and selected components of the WCST,^28,30,66^ we identified multiple lncRNAs associated with performance in memory, language, executive function, and attention. These findings support the emerging view that circulating lncRNAs reflect molecular processes relevant to AD pathophysiology and may contribute to cognitive decline.

We detected significant correlations between lncRNAs and 12 of the 25 cognitive variables assessed. Associations were particularly notable for the BNT, where lncRNAs mapping to *EPHB1*, *DLEU1*, and *CHRNA4* correlated positively with spontaneous cue generation and correct responses, while ENST00000565094 (*HEATR4*) showed negative associations. Additional significant correlations were observed across ROCFT, Stroop, TMT, Token, and WCST tasks, underscoring the broad cognitive relevance of these transcripts (Table 2).

Several mapped genes—*FIRRE*, *TEAD1*, *VDAC3*, *PRDM2*, *FBXL2*, *KCNJ14*, *LY6E*-*DT*, *PPT2*-*EGFL8*, *EMX2OS*, *BCAS3*, and *FAM221A*—play roles in synaptic signaling, cholinergic neurotransmission, mitochondrial regulation, and neuroinflammation.^1,67^ These biological pathways are severely disrupted in AD and align with our observed cognitive associations.

*EPHB1*, a member of the Eph receptor family, supports synaptic maintenance, glutamatergic signaling, and neuroinflammatory modulation. Altered Eph signaling and mutations linked to synaptic dysfunction have been documented in AD.^68–76^

*CHRNA4*, which encodes the α4 subunit of the α4β2 nicotinic receptor, was also associated with language measures. Reductions in α4β2 receptors occur in AD,^77^ and CHRNA4 variants have been linked to oxidative injury and Aβ accumulation.^78,79^ Prior work highlights its role in attention, memory, and neural connectivity.^80,81^

*TEAD1* participates in neuronal differentiation, proliferation, and survival, and its dysregulation may affect neuroprotective and immune-related mechanisms relevant to AD.^82–84^ *VDAC3*, associated with ROCFT performance, contributes to mitochondrial homeostasis and oxidative stress responses—central features of AD pathology.^85–87^

Other high-priority predictive genes included *APP*, *POT1*, *SLC8A1*, *EEA1*, and *IBA57*. These genes participate in protein homeostasis, DNA stability, mitochondrial metabolism, and neuroinflammatory signaling. For example, *SLC8A1* regulates neuronal calcium homeostasis and has been implicated in ferroptosis and inflammation.^88–91^ *ZNF426-DT* also emerged as relevant across several cognitive domains, with putative roles in transcriptional regulation and oxidative stress.^92^

A critical challenge in biomarker studies involving pooled cohorts is the potential for “group-driven” correlations, where molecular differences might appear to be driven primarily by the AD status rather than a continuous relationship with clinical severity. To address this, we employed the PPS (Table 7), a non-linear, asymmetric metric that evaluates predictive relationships more rigorously than traditional linear models.^61,62^ By using decision-tree-based evaluations, the PPS identifies lncRNAs whose expression levels provide genuine information about cognitive performance across the entire cohort. Furthermore, our within-group analyses and interaction models (Supplemental Table 7 and Supplemental Figures 1–12) confirmed that the identified lncRNA signatures remain informative of clinical severity even when restricted to the AD group. This suggests that these transcripts are not merely markers of the disease state but are actively reflecting the biological progression and the cognitive architecture of the neurodegenerative process.

This study benefits from its combined use of detailed cognitive phenotyping and high-resolution transcriptomic profiling, allowing a biologically informed interpretation of neuropsychological performance. The multi-method prioritization strategy increases confidence in the identified lncRNA candidates.

Nevertheless, several limitations should be acknowledged. First, AD diagnosis in our cohort was established based on clinical rather than the biological Amyloid, Tau, and Neurodegeneration (ATN) framework.^93^ Due to regional resource constraints in Colombia at the time of the study, confirmation via amyloid/tau PET imaging or CSF biomarkers was not available. The absence of biological confirmation represents a significant limitation, as clinical diagnosis alone may lead to the inclusion of mixed etiologies.^94^ Consequently, while our findings highlight lncRNA signatures strongly associated with cognitive decline and clinical phenotypes, their specificity to pure AD pathology remains to be validated in future cohorts with confirmed amyloid and tau status.

A second limitation is the relatively small sample size (*n* = 30) in comparison to the high-dimensional nature of the microarray-scale features (*p* > 29,000). This “large *p*, small *n*” scenario inherent to high-throughput transcriptomics inherently limits statistical power and generalizability, increasing the risk of unstable findings or overfitting.^95^ To mitigate this, we employed the Bonferroni procedure to control the Type I error. While biologically plausible candidate lncRNAs were identified, these results should be interpreted as hypothesis-generating.^96^ Consequently, our findings represent a discovery phase that requires independent replication and experimental functional validation. Furthermore, while blood-derived exosomal RNA serves as a minimally invasive and promising biomarker source, it may not fully mirror the absolute brain-specific expression profiles due to the blood-brain barrier and systemic RNA contributions.

Future research should include longitudinal sampling, integration with genomic, proteomic, and metabolomic datasets, and functional testing in cellular or animal models. Evaluating lncRNAs in other accessible fluids such as CSF may further enhance biomarker development. Therapeutic strategies targeting lncRNAs, such as antisense oligonucleotide approaches, represent a promising avenue requiring further exploration.

### Conclusions

This study advances understanding of the complex molecular landscape underlying neuropsychological impairment in AD by identifying lncRNAs significantly associated with cognitive performance across multiple domains. Our findings support the emerging view of lncRNAs as key players in AD pathogenesis and highlight their potential as biomarkers and therapeutic targets.

Given the cross-sectional design of this study, it is important to note that the observed relationships between lncRNA expression and cognitive performance are correlational. Hence, we cannot conclude whether these transcriptomic changes precede or follow the onset of clinical symptoms. Further longitudinal studies and functional in vitro experiments are required to elucidate whether these lncRNAs are active drivers of neurodegeneration or reactive products of the disease process. Furthermore, continued research into lncRNA biology will be essential for developing novel diagnostic tools and personalized interventions to better understanding and combating this devastating disease.

## Supplemental Material

sj-docx-1-alz-10.1177_13872877261470430 - Supplemental material for Novel blood lncRNA biomarkers associated with clinical severity and specific cognitive dimensions in Alzheimer's disease Supplemental material, sj-docx-1-alz-10.1177_13872877261470430 for Novel blood lncRNA biomarkers associated with clinical severity and specific cognitive dimensions in Alzheimer's disease by María I. Mosquera-Heredia, Oscar M. Vidal, Ernesto Barceló, Luis C. Morales, Carlos Silvera-Redondo, Daniel A. Bolívar, Ricardo Allegri, Mauricio Arcos-Burgos, Pilar Garavito-Galofre and Jorge I. Vélez in Journal of Alzheimer's Disease

sj-xlsx-2-alz-10.1177_13872877261470430 - Supplemental material for Novel blood lncRNA biomarkers associated with clinical severity and specific cognitive dimensions in Alzheimer's disease Supplemental material, sj-xlsx-2-alz-10.1177_13872877261470430 for Novel blood lncRNA biomarkers associated with clinical severity and specific cognitive dimensions in Alzheimer's disease by María I. Mosquera-Heredia, Oscar M. Vidal, Ernesto Barceló, Luis C. Morales, Carlos Silvera-Redondo, Daniel A. Bolívar, Ricardo Allegri, Mauricio Arcos-Burgos, Pilar Garavito-Galofre and Jorge I. Vélez in Journal of Alzheimer's Disease

sj-xlsx-3-alz-10.1177_13872877261470430 - Supplemental material for Novel blood lncRNA biomarkers associated with clinical severity and specific cognitive dimensions in Alzheimer's disease Supplemental material, sj-xlsx-3-alz-10.1177_13872877261470430 for Novel blood lncRNA biomarkers associated with clinical severity and specific cognitive dimensions in Alzheimer's disease by María I. Mosquera-Heredia, Oscar M. Vidal, Ernesto Barceló, Luis C. Morales, Carlos Silvera-Redondo, Daniel A. Bolívar, Ricardo Allegri, Mauricio Arcos-Burgos, Pilar Garavito-Galofre and Jorge I. Vélez in Journal of Alzheimer's Disease

sj-xlsx-4-alz-10.1177_13872877261470430 - Supplemental material for Novel blood lncRNA biomarkers associated with clinical severity and specific cognitive dimensions in Alzheimer's disease Supplemental material, sj-xlsx-4-alz-10.1177_13872877261470430 for Novel blood lncRNA biomarkers associated with clinical severity and specific cognitive dimensions in Alzheimer's disease by María I. Mosquera-Heredia, Oscar M. Vidal, Ernesto Barceló, Luis C. Morales, Carlos Silvera-Redondo, Daniel A. Bolívar, Ricardo Allegri, Mauricio Arcos-Burgos, Pilar Garavito-Galofre and Jorge I. Vélez in Journal of Alzheimer's Disease

sj-xlsx-5-alz-10.1177_13872877261470430 - Supplemental material for Novel blood lncRNA biomarkers associated with clinical severity and specific cognitive dimensions in Alzheimer's disease Supplemental material, sj-xlsx-5-alz-10.1177_13872877261470430 for Novel blood lncRNA biomarkers associated with clinical severity and specific cognitive dimensions in Alzheimer's disease by María I. Mosquera-Heredia, Oscar M. Vidal, Ernesto Barceló, Luis C. Morales, Carlos Silvera-Redondo, Daniel A. Bolívar, Ricardo Allegri, Mauricio Arcos-Burgos, Pilar Garavito-Galofre and Jorge I. Vélez in Journal of Alzheimer's Disease

sj-xlsx-6-alz-10.1177_13872877261470430 - Supplemental material for Novel blood lncRNA biomarkers associated with clinical severity and specific cognitive dimensions in Alzheimer's disease Supplemental material, sj-xlsx-6-alz-10.1177_13872877261470430 for Novel blood lncRNA biomarkers associated with clinical severity and specific cognitive dimensions in Alzheimer's disease by María I. Mosquera-Heredia, Oscar M. Vidal, Ernesto Barceló, Luis C. Morales, Carlos Silvera-Redondo, Daniel A. Bolívar, Ricardo Allegri, Mauricio Arcos-Burgos, Pilar Garavito-Galofre and Jorge I. Vélez in Journal of Alzheimer's Disease

sj-xlsx-7-alz-10.1177_13872877261470430 - Supplemental material for Novel blood lncRNA biomarkers associated with clinical severity and specific cognitive dimensions in Alzheimer's disease Supplemental material, sj-xlsx-7-alz-10.1177_13872877261470430 for Novel blood lncRNA biomarkers associated with clinical severity and specific cognitive dimensions in Alzheimer's disease by María I. Mosquera-Heredia, Oscar M. Vidal, Ernesto Barceló, Luis C. Morales, Carlos Silvera-Redondo, Daniel A. Bolívar, Ricardo Allegri, Mauricio Arcos-Burgos, Pilar Garavito-Galofre and Jorge I. Vélez in Journal of Alzheimer's Disease

## Data Availability

The data presented in this study and scripts used to derive our findings are available from the corresponding author upon reasonable request. They are not publicly available due to the ongoing nature of the study and our commitment to protecting the privacy and confidentiality of our patients.
