# Supplementary material for: Novel blood lncRNA biomarkers associated with clinical severity and specific cognitive dimensions in Alzheimer's disease
Source: J Alzheimers Dis. 2026 Jul 29;113(2):701–20. doi: 10.1177/13872877261470430 (PMC13691238; doi:10.1177/13872877261470430)
Supplement: Supplementary file 1 [file sj-docx-1-alz-10.1177_13872877261470430.docx]

Supplemental Material

Novel blood lncRNA biomarkers associated with clinical severity and specific cognitive dimensions in Alzheimer’s disease

**RNA isolation**

A total of 6 mL of blood was obtained by conventional venipuncture in tubes, without additive, for each participant.^1–3^ The tubes were centrifuged at 4000 RPM for 10 minutes to obtain serum. The serum was centrifuged at 5000 RPM for 30 minutes to remove any remaining vesicles or detritus. The supernatant was subsequently transferred without disturbing the pellet to a new Eppendorf tube and placed on conventional ice until use.

The Total Exosome Isolation Reagent commercial kit (catalog #4478360, Thermo Fisher Scientific, Inc., USA) was used to isolate the exosomes following the manufacturer’s instructions with minor modifications standardized at the laboratories of Universidad del Norte, Barranquilla; 1,000 µL of serum and 200 µL of the reagent were transferred to an Eppendorf tube. This mixture was homogenized in a vortex for 1 minute to obtain a cloudy solution.^1–3^ This mixture was subsequently incubated at -20^o^C for 30 minutes at rest and in a vertical position, taking care not to mix during or after incubation. Later, the mixture was then centrifuged at 10000 RPM for 30 minutes at room temperature. The supernatant was aspirated with micropipette and discarded in order not to alter the pellet since the exosomes are found there. The pellet was resuspended adding 200 µL of PBS 1X and thus ready for further studies. Resulting exosomes were characterized by scanning electron microscopy (SEM). For this purpose, exosomes were encapsulated with nanodiamond particles, and their sizes were meticulously confirmed, with measurements revealing a maximum diameter of 160 nm.^1–3^

**Exosomal RNA extraction**

For the extraction of RNA contained in exosomes, a technique based on the acid phenol:chloroform method was standardized in the laboratory of the Universidad del Norte.^1–3^ To resuspended exosomes (200 µL), we added 200 µL of a denaturing solution 2X and proceeded to vortex mix for 1 minute.^1–3^ Then, this mixture was incubated at -20^o^C for 5 minutes adding 400 µL phenol:chloroform acid, and subsequently vortexed for 1 minute and centrifuged for 13 minutes at 12,000 RPM at room temperature to separate the mixture into aqueous and organic phases. This step was repeated when the interface was not compact.

Further, the aqueous (upper) phase was carefully removed without disturbing the lower phase and the interphase and transferred to a new tube. Considering the volume recovered, 80% isopropanol and 20% 3M sodium acetate pH 5.2 were added and homogenized. The sample was frozen overnight at -20^o^C in an upright position, taking care not to mix during or after freezing.^1–3^ At the end of the incubation time, the mixture was centrifuged at 14000 RPM for 10 minutes at room temperature, discarding the supernatant by inversion. Seventy-five percent ethanol (two volumes with respect to the amount of isopropanol/sodium acetate used) was added to the pellet and centrifuged at 10000 RPM for 10 minutes at room temperature. The supernatant was discarded by inversion, and the tubes were allowed to dry upside down for 10 minutes.^1–3^ Extracted RNA was resuspended with 50 µL of RNAsase-free water and then subjected to DNase I (catalog #EN0521, Thermo Fisher Scientific, Inc., USA) following the manufacturer's instructions. Finally, the concentration and indexes of the readings obtained with the optical densities (OD) 260/230 y 260/280 were measured in a NanoDrop 2000 (Thermo Fisher Scientific, Inc., USA), and correspond to the RNA quality indexes.

**lncRNA microarray study**

For lncRNA identification and differential expression analysis, the 30 samples (15 cases with AD and 15 healthy controls) were sent to Arraystar, Inc (Rockville, MD USA; <https://www.arraystar.com/>), where RNA quality control, as well as complementary RNA (cRNA) synthesis, labeling and hybridization were performed according to Agilent’s single-color microarray-based gene expression analysis protocol (Agilent Technologies, USA) with minor modifications.^1^

*Quality control*

Before starting the microarray study, the quality, purity and concentration of the exosomal RNA samples obtained were corroborated.^1^ To establish that the RNA is pure, the OD260/280 ratio should be close to 2.0, while the OD260/230 ratio must be >1.8. RNA integrity was assessed by denaturing agarose gel electrophoresis to detect clear bands of ribosomal RNA (rRNA) 28S and 18S with a 28S:18S intensity ratio close to 2:1, which indicates that RNA is intact.^1^

*Complementary RNA synthesis and tagging*

Each sample was subjected to retrotranscription to obtain complementary DNA (cDNA); this was amplified and transcribed back to its com-plementary RNA (cRNA).^1^ In this step, amplification and incorporation of the cyanine 3 (Cy3) fluorescent dye labeling is achieved simultaneously along the entire length of the 3' unbiased transcript using a random priming method (Arraystar Flash RNA Labelling Kit, Arraystar, Inc., Rockville, MD, USA). The labelled cRNAs were purified with the RNeasy mini kit (Qiagen, Hilden, Germany). In this step it was possible to eliminate reagent residues and the excess of cyanine not incorporated. As a control of the amplification and labelling process of the samples, the concentration of the cRNA obtained and the rate of cyanine incorporation or specific activity (pmol of Cy3 per μg cRNA). Hybridization was allowed to continue if the cRNA concentration was >1.65 μg and the specific activity was >9 pmol de Cy3 per μg of cRNA. Otherwise, cRNA preparation was repeated.^1^

*Hybridization and microarray scanning*

A total of 1 μg of each labeled cRNA was fragmented by the addition of 5 μL of blocking agent 10X and 1 μL of fragmentation buffer 25X.^1^ The mixture was heated to 60°C for 30 minutes, and then added 25 μL of hybridization buffer 2X GE to dilute the labelled cRNA; 50 μL of hybridization solution were dispensed onto a hybridization plate which was then assembled with a lncRNA expression microarray plate. The plates were incubated for 17 hours at 65°C in an Agilent hybridization oven. The hybridized arrays were washed and then scanned using an Agilent scanner (equipment #G2505C, USA).^1^

*lncRNA microarray and data normalization*

The Arraystar Human LncRNA Microarray v5.0 was used in this study.^1^ This microarray quantifies the expression of 39317 lncRNAs (8393 gold standard lncRNAs and 30924 reliable lncRNAs). Arraystar, Inc has high-quality, proprietary lncRNA transcriptome databases, which compile lncRNAs through major public databases and repositories. Each transcript is identified with a splice probe. In this case, 60491 probes of 60 nt length were used. For hybridization quality control, positive probes and negative probes for domestic genes, designed by the company, were used. Hybridization signals on the microarray were read using the Agilent’s Feature Extraction software, version 11.0.1.1. Data were normalized using quantile normalization^4^ and expression values were adjusted using a linear model as implemented in GeneSpring GX v12.1 (Agilent Technologies).

*Identification of differentially expressed lncRNAs*

After normalization, lncRNAs that were flagged as present or marginal (“all-target value”) in at least 15 out of 30 samples were chosen.^1^ Differentially expressed lncRNAs between cases and controls were determined based on the *p-*value of a two-sample *t*-test. To control false positives, these *p*-values were corrected using the false discovery rate (FDR).^5,6^ Thus, FDR-corrected *p-*values below a Type I error of 5% (*p*_FDR_ < 0.05) were considered statistically significant. lncRNAs were also filtered using a Fold Change [FC] $\geq$1.5.

*lncRNA annotation*

Functional analysis of differentially expressed exosomal lncRNAs was performed using proprietary, high-quality transcriptome and lncRNA databases.^1^ Thus, information was obtained on lncRNA transcription unit identifications, including their length (number of nucleotides), gene identifications and symbols, loci, positions in relation to neighboring protein-coding genes (intronic or exonic overlap, sense, antisense, bidirectional or whether they are intergenic transcripts), functional molecular mechanisms, association with cells or tissues and subcellular locations^1^. In addition, scientific publications in the Web of Science, PubMed and SCOPUS databases were continuously reviewed and selected, as well as the following databases for the annotation of results: LncRNAdb v2.0 (<http://www.lncrnadb.org/>), RNAdb v2.0 (<http://research.imb.uq.edu.au/rnadb/>), GENCODE v21 (<https://www.gencodegenes.org>), RefSeq (<https://www.ncbi.nlm.nih.gov/refseq/>), ENCODE CAGE (<https://www.encodeproject.org>), UCSC_knowngene (<https://genome.ucsc.edu/cgi-bin/hgTrackUi?db=hg38&g=knownGene&c=chrX>), FANTOM5CAT (<https://fantom.gsc.riken.jp/5/data/>), LncBook (<https://ngdc.cncb.ac.cn/lncbook/>), UCSC Genome Browser (<https://genome.ucsc.edu/cgi-bin/hgGateway>), MalaCards (<https://www.malacards.org>), GeneCards (<https://www.genecards.org>), KEGG (<https://www.genome.jp/kegg/kegg2.html>), STRING V11.5. (<https://string-db.org/cgi/input?sessionId=blmgf96LnfJ8&input_page_show_search=on>), Human Genome Connectome (HGC; <https://hgc.rockefeller.edu/>) and AlzGene (<http://www.alzgene.org/>).

**Supplemental Tables 1-6 are available as separate Excel file.**

**Supplemental Table 7.** Validation of lncRNA-cognitive associations via within-group and interaction modelling. This table presents a detailed statistical evaluation of the lncRNAs reported in Table 2 of the main manuscript. To distinguish between-group separation from true clinical severity signals, we report: (*i*) within-group associations restricted to the Alzheimer’s disease (AD) cohort, and (*ii*) interaction models testing the relationship between lncRNA expression and cognitive performance across diagnoses (lncRNA x AD diagnosis. *p-*values were adjusted for age, sex, and education and corrected for multiple testing using Bonferroni’s method. Overall, these analyses confirm the consistency of the molecular-cognitive associations within the pathological group.

| **Neuropsychological test** | **Transcript** | **Chr** | **Position** | **Closest gene** | **Statistically significant?** | |
| --- | --- | --- | --- | --- | --- | --- |
|  |  |  |  |  | **AD group** | **Interaction** |
| *BNT* |  |  |  |  |  |  |
| Spontaneous | T367347 | 9 | 131,975,444 | *RP11-247A12.2* | Yes | Yes |
|  | ENST00000488154 | 3 | 134,514,474 | *EPHB1* | Yes | Yes |
|  | ENST00000606683 | 5 | 139,024,045 | *CTD-3224K15.3* | No | Yes |
|  | ENST00000565094 | 14 | 73,945,189 | *HEATR4* | No | Yes |
|  | TCONS_00024398 | 16 | 51,558,114 | *HNRNPA1P48* | Yes | Yes |
|  | ENST00000413708 | 2 | 107,978,775 | *AC006227.1* | Yes | Yes |
|  | ENST00000486895 | 13 | 50,656,307 | *DLEU1* | Yes | Yes |
|  | ENST00000370257 | 20 | 61,991,340 | *CHRNA4* | No | Yes |
|  | ENST00000503593 | 4 | 146,998,998 | *RP11-203B7.1* | Yes | Yes |
|  | NR_125739 | 20 | 48,801,140 | *CEBPB* | Yes | Yes |
| Total | ENST00000488154 | 3 | 134,514,474 | *EPHB1* | Yes | Yes |
|  | T367347 | 9 | 131,975,444 | *RP11-247A12.2* | Yes | Yes |
|  | ENST00000565094 | 14 | 73,945,189 | *HEATR4* | No | Yes |
|  | ENST00000606683 | 5 | 139,024,045 | *CTD-3224K15.3* | Yes | Yes |
|  | ENST00000370257 | 20 | 61,991,340 | *CHRNA4* | Yes | Yes |
|  | NR_125739 | 20 | 48,801,140 | *CEBPB* | Yes | Yes |
|  | TCONS_00022695 | 14 | 29,530,332 | *RP11-148E17.1* | Yes | Yes |
|  | TCONS_00024398 | 16 | 51,558,114 | *HNRNPA1P48* | Yes | Yes |
|  | ENST00000606343 | 1 | 156,607,575 | *RP11-284F21.9* | No | Yes |
|  | ENST00000413708 | 2 | 107,978,775 | *AC006227.1* | Yes | Yes |
| *ROCFT* |  |  |  |  |  |  |
| Copy | ENST00000427391 | X | 130,836,680 | *FIRRE* | No | No |
|  | ENST00000595232 | 3 | 106,991,514 | *LINC00883* | No | No |
|  | ENST00000419640 | 2 | 192,093,891 | *AC092614.2* | No | No |
|  | ENST00000528792 | 11 | 12,870,342 | *TEAD1* | No | No |
|  | T343001 | 8 | 42,260,903 | *VDAC3* | No | No |
|  | ENST00000512200 | 11 | 68,638,132 | *RP11-757G1.6* | No | No |
|  | ENST00000531347 | 11 | 69,918,540 | *ANO1-AS2* | No | Yes |
|  | ENST00000569677 | 16 | 79,246,608 | *RP11-679B19.2* | No | Yes |
|  | MICT00000302088 | 6 | 33,945,117 | *MIR1275* | No | No |
|  | ENST00000476738 | 13 | 50,656,307 | *DLEU1* | No | No |
| Recall | ENST00000438699 | 9 | 66,523,532 | *RP11-262H14.3* | No | No |
|  | T260896 | 4 | 8,645,137 | *CPZ* | No | No |
|  | T003446 | 1 | 14,152,137 | *PRDM2* | No | No |
|  | ENCT00000301655 | 3 | 33,316,741 | *FBXL2* | No | No |
|  | ENST00000596497 | 19 | 48,968,865 | *KCNJ14* | No | No |
|  | ENST00000491609 | X | 134,022,984 | *MOSPD1* | No | No |
|  | TCONS_00016056 | 9 | 91,825,614 | *SHC3* | No | No |
|  | ENST00000649444 | 5 | 40,463,664 | *AC108105.1* | No | No |
| *Stroop test* |  |  |  |  |  |  |
| Colours | TCONS_00028265 | 20 | 62,030,713 | *KCNQ2* | No | Yes |
|  | ENST00000512200 | 11 | 68,638,132 | *RP11-757G1.6* | No | No |
|  | ENST00000502167 | 8 | 144,089,170 | *LY6E-DT* | No | No |
|  | ENST00000489721 | 6 | 32,135,320 | *PPT2-EGFL8* | No | No |
|  | ENST00000440007 | 10 | 119,243,806 | *EMX2OS* | No | No |
|  | ENST00000577456 | 22 | 20,632,023 | *AC011718.2* | No | No |
|  | ENST00000434437 | 6 | 138,266,503 | *RP11-10J5.1* | No | No |
|  | T172585 | 19 | 30,265,492 | *CCNE1* | No | No |
|  | ENST00000572864 | 17 | 45,148,812 | *RP11-156P1.3* | No | No |
|  | NR_125764 | 3 | 164,924,748 | *RP11-85M11.2* | No | No |
| Words | ENST00000448272 | 10 | 13,457,202 | *RP11-445K13.2* | No | No |
|  | ENST00000601901 | 20 | 26,168,268 | *MIR663A* | No | No |
|  | ENCT00000408932 | 7 | 12,725,221 | *CTD-2320J21.1* | No | No |
|  | T247315 | 3 | 103,841,735 | *RP11-40M23.1* | No | No |
|  | ENST00000512200 | 11 | 68,638,132 | *RP11-757G1.6* | No | No |
|  | ENST00000603624 | 9 | 138,354,573 | *PPP1R26-AS1* | No | No |
|  | ENST00000502167 | 8 | 144,089,170 | *LY6E-DT* | No | No |
|  | ENST00000616315 | 11 | 65,193,022 | *NEAT1* | No | No |
|  | ENST00000649005 | 14 | 53,258,503 | *GNPNAT1* | No | No |
|  | ENST00000454029 | 7 | 23,719,814 | *FAM221A* | No | No |
| *TMT* |  |  |  |  |  |  |
| Part A | ENST00000585495 | 17 | 59,460,762 | *BCAS3* | No | No |
| Part B | ENST00000590478 | 17 | 33,558,469 | *RP11-799D4.4* | No | No |
|  | ENST00000446238 | 1 | 75,593,515 | *RP11-510C10.2* | No | No |
|  | ENST00000445226 | 1 | 44,410,044 | *RP11-7O11.3* | No | No |
|  | T323724 | 7 | 49,168,842 | *AC010971.1* | No | No |
| Token test | GSE61474_TCONS_00156017 | 18 | 68,132,153 | *RPS2P6* | No | Yes |
|  | TCONS_l2_00001470 | 1 | 118,359,347 | *AL390877.1* | No | Yes |
|  | TCONS_00020904 | 12 | 105,836,748 | *C12orf75* | No | No |
| *WCST* |  |  |  |  |  |  |
| Correct answers | ENST00000543558 | 12 | 119,083,120 | *RP11-121J20.1* | Yes | Yes |
|  | ENST00000505626 | 5 | 6,582,251 | *LINC01018* | Yes | Yes |
|  | ENST00000592775 | 6 | 146,136,434 | *RP11-545I5.3* | Yes | Yes |
|  | ENST00000445551 | 1 | 47,897,805 | *FOXD2-AS1* | Yes | Yes |
|  | T198116 | 2 | 121,876,102 | *AC067960.1* | Yes | Yes |
|  | ENST00000373508 | 20 | 36,305,312 | *RP4-640H8.2* | Yes | Yes |
|  | T119156 | 15 | 72,891,278 | *RP11-361M10.3* | Yes | Yes |
|  | HSALNT0289277 | 20 | 48,657,243 | *TRERNA1* | Yes | Yes |
|  | ENST00000482799 | 7 | 98,628,218 | *SMURF1* | Yes | Yes |
|  | ENST00000463529 | X | 48,755,793 | *PQBP1* | Yes | Yes |
| NPE | ENST00000426962 | 2 | 242,913,429 | *AC131097.3* | No | No |
|  | ENST00000602483 | 4 | 119,199,910 | *SNHG8* | No | No |
|  | ENST00000593211 | 19 | 32,413,790 | *CTC-360P9.5* | No | No |
|  | ENST00000521563 | 5 | 139,483,425 | *MALINC1* | No | No |
|  | NR_125345 | 19 | 52,892,093 | *ZNF528-AS1* | No | No |
|  | TCONS_00015892 | 9 | 137,419,027 | *RP11-473E2.4* | No | Yes |
|  | ENST00000638682 | 17 | 33,895,128 | *RP11-1094M14.11* | No | No |
|  | T007806 | 1 | 35,497,914 | *ZMYM6* | No | Yes |
|  | ENST00000532068 | 11 | 45,755,545 | *CTD-2210P24.1* | No | Yes |
|  | HBMT00000551071 | 16 | 84,651,102 | *COTL1* | No | No |
| Perseverant errors | T209245 | 2 | 230,613,416 | *TRIP12* | Yes | No |
|  | ENCT00000088265 | 12 | 12,420,309 | *LRP6* | Yes | No |
|  | ENST00000479646 | 20 | 36,888,608 | *KIAA1755* | Yes | No |
|  | FTMT25600002825 | 14 | 65,347,312 | *SPTB* | Yes | No |
|  | TCONS_00019769 | 11 | 115,754,859 | *AP000797.3* | Yes | No |
|  | ENCT00000408932 | 7 | 12,725,221 | *CTD-2320J21.1* | Yes | No |
|  | ENST00000608183 | 1 | 182,098,445 | *GS1-122H1.2* | Yes | Yes |
|  | T152907 | 17 | 65,673,974 | *PITPNC1* | Yes | No |
|  | T286073 | 5 | 92,934,175 | *NR2F1* | Yes | No |
|  | ENST00000511243 | 11 | 96,180,296 | *JRKL-AS1* | Yes | No |

**Supplemental Figure 1.** Correlation between lncRNA expression and BNT (spontaneous).

**Supplemental Figure 2.** Correlation between lncRNA expression and BNT (total).

**Supplemental Figure 3.** Correlation between lncRNA expression and ROCFT (Copy).

**Supplemental Figure 4.** Correlation between lncRNA expression and ROCFT (Recall).

**Supplemental Figure 5.** Correlation between lncRNA expression and Stroop (colors).

**Supplemental Figure 6.** Correlation between lncRNA expression and Stroop (words).

**Supplemental Figure 7.** Correlation between lncRNA expression and TMT (Part A).

**Supplemental Figure 8.** Correlation between lncRNA expression and TMT (Part B).

**Supplemental Figure 9.** Correlation between lncRNA expression and Token test.

**Supplemental Figure 10.** Correlation between lncRNA expression and WCST (correct answers).

**Supplemental Figure 11.** Correlation between lncRNA expression and WCST (non-perseverant errors).

**Supplemental Figure 12.** Correlation between lncRNA expression and WCST (perseverant errors).

**References**

1.	Mosquera-Heredia MI, Vidal OM, Morales LC, et al. Long non-coding RNAs and Alzheimer’s disease: towards personalized diagnosis. *Int J Mol Sci* 2024; 25: 7641.

2.	Bolívar DA, Mosquera-Heredia MI, Vidal OM, et al. Exosomal mRNA signatures as predictive biomarkers for risk and age of onset in Alzheimer’s disease. *Int J Mol Sci* 2024; 25: 12293.

3.	Barceló E, Mosquera-Heredia MI, Vidal OM, et al. Deficits of Alzheimer’s disease neuropsychological architecture correlate with specific exosomal mRNA expression: evidence of a continuum? *Int J Mol Sci* 2025; 26: 4897.

4.	Bolstad BM, Irizarry RA, Astrand M, et al. A comparison of normalization methods for high density oligonucleotide array data based on variance and bias. *Bioinformatics* 2003; 19: 185–93.

5.	Benjamini Y and Hochberg Y. Controlling the false discovery rate - a practical and powerful approach to multiple testing. *J R Stat Soc Series B (Methodol)* 1995; 57: 289–300.

6.	Vélez JI, Correa JC and Arcos-Burgos M. A new method for detecting significant p-values with applications to genetic data. *Rev Colomb Estad* 2014; 37: 67–76.
